# Digital Health Use and Interest in Virtual Cardiac Rehabilitation Among Cardiac Inpatients: Cross-Sectional Study

**DOI:** 10.2196/91767

**Published:** 2026-09-11

**Authors:** Hamza G Patel, Jie Ding, Xinyi Zhang, Abel Cherian, Pauline Hyunh, Apurva Sharma, Vennela Avula, Rongzi Shan, Katherine A Ornstein, Shireen R Khoury, Francoise A Marvel, Lena M Mathews, Seth S Martin, Erin M Spaulding

**Affiliations:** 1 Division of Cardiology Department of Medicine, Johns Hopkins Medicine, Baltimore, MD, United States; 2 Digital Health Innovation Laboratory Ciccarone Center for the Prevention of Cardiovascular Disease, Division of Cardiology, Department of Medicine, Johns Hopkins University School of Medicine, Baltimore, MD, United States; 3 School of Nursing, Johns Hopkins University, 525 North Wolfe Street, Baltimore, MD, 21205, United States, 410-955-4766; 4 University of Arizona College of Medicine-Tucson/Banner University Medical Center, Tucson, AZ, United States; 5 Kaiser Permanente Oakland Medical Center, Oakland, CA, United States; 6 Smidt Heart Institute Department of Cardiology, Cedars-Sinai Medical Center, Los Angeles, CA, United States; 7 Center for Health Equity, Johns Hopkins University, Baltimore, MD, United States; 8 Welch Center for Prevention, Epidemiology, and Clinical Research, Johns Hopkins University, Baltimore, MD, United States; 9 Whiting School of Engineering, Johns Hopkins University, Baltimore, MD, United States

**Keywords:** digital health, telemedicine, virtual cardiac rehabilitation, cardiac rehabilitation, mobile health

## Abstract

**Background:**

Cardiac rehabilitation (CR) improves patient quality of life, morbidity, and mortality. Unfortunately, it is underused by patients. Digital health interventions offer a solution to increase participation in CR. However, patients’ interest in virtual CR, especially among those in the inpatient setting, has not been fully explored. The benefits of CR have been predominantly demonstrated in traditional, center-based CR programs.

**Objective:**

The objective of this cross-sectional study with prospective enrollment was to explore inpatient interest in virtual CR among adult patients who were hospitalized with a CR-qualifying diagnosis.

**Methods:**

A Qualtrics survey comprising multiple-choice questions was administered to cardiac inpatients at the progressive cardiac care unit at the Johns Hopkins Hospital from January 2020 to March 2024. Sociodemographic and clinical characteristics were retrieved from the electronic medical records. The study included English-speaking patients aged 18 years or older with a diagnosis eligible for CR. Digital health use was compared between an earlier enrollment period (2020‐2022) and a later enrollment period (2023‐2024).

**Results:**

A total of 150 patients were included (mean age 64, SD 13 years; n=57, 38% female, and n=85, 56.7% White). With respect to sociodemographic characteristics, 26% (n=39) of the patients had a high school education or less, 53.8% (70/130) were married, 26.2% (34/130) were employed full time, and 63.8% (83/130) had private insurance. In exploratory enrollment-period comparisons, texting health care providers was more commonly reported in 2023 to 2024 than in 2020 to 2022 (74/123, 60.2% vs 6/27, 22.2%; *P*<.001), as was using a smartphone or tablet to learn about illnesses (93/123, 75.6% vs 17/27, 63%; *P*=.04) and to discuss health with a physician or nurse practitioner (86/123, 69.9% vs 12/27, 44.4%; *P*=.01). Participants with more than a high school education were more likely to perceive smartphones as beneficial for leading a healthier lifestyle (50/104, 48.1% vs 9/37, 24.3%, *P*=.01) and learning about illnesses (90/105, 85.7% vs 20/37, 54.1%, *P*<.001) than were participants with a high school education or less. Participants across all sociodemographic factors expressed interest in virtual CR (overall 107/150, 71.3%), with non-White participants being more interested than White participants (55/65, 84.6% vs 52/85, 61.2%, *P*=.002).

**Conclusions:**

Most cardiac inpatients expressed interest in home-based or virtual CR to alleviate barriers to in-person CR participation. Future work should emphasize digital equity and user support to optimize the widespread adoption of virtual CR.

## Introduction

There are many effective interventions for cardiovascular disease (CVD) that resolve immediate risk and symptoms in patients. However, the modifiable risk factors that predispose patients to developing CVD in the first place remain to be fully addressed, and CVD remains the most significant cause of mortality worldwide [1]. Cardiac rehabilitation (CR) bridges the gap in the transition from acute interventions to long-term preventative care [2]. CR is a multidisciplinary intervention that includes health education and supervised physical activity tailored to the individual. This reduces the burden of CVD by enhancing the patient’s recovery from an acute cardiac event and contributing to lasting lifestyle modifications. Many studies have demonstrated the benefits of CR, predominantly in traditional, center-based CR programs, including its ability to improve cardiovascular health and quality of life as well as decrease mortality and hospitalizations [3,4].

Despite this strong evidence, CR is widely underutilized by patients, with attendance rates in the United States ranging from 19% to 34% in national analyses [5,6]. Additionally, the 2019 United Kingdom National Audit of Cardiac Rehabilitation revealed that older individuals, women, patients with multiple comorbidities, patients in rural communities, and ethnic minority groups are much less likely to attend CR than others [7]. This lack of use is due to a variety of interrelated factors at the patient, clinician, and health care levels. At the patient level, the cost of traveling to the CR program, ease of accessibility, time away from work, and lack of motivation, among other reasons, tend to be common concerns that prevent patients from engaging in CR [4]. At the clinician level, a lack of introduction to CR in medical training could translate to an inability to properly emphasize the importance of CR during patient education [4]. Even when referral occurs, however, referral alone may be insufficient to ensure patient participation. Referral rates to CR programs by physicians increased to approximately 80% in more recent studies among patients after acute myocardial infarction, but less than half of the patients attended [5,8]. Finally, at the health care level, this lack of use of CR by patients could be due to reduced capacities, temporary closure of centers, and lack of reimbursement and resources [9,10].

These system-level barriers became particularly visible during the COVID-19 pandemic, when in-person, group-based CR sessions were disrupted by infection-control measures and shelter-in-place policies [9,10]. However, the pandemic also gave rise to the need for the extension of CR services to the home and to new avenues of delivering home-based CR to patients by leveraging technology, such as smartphones and wearable devices [9-12].

Much of the focus on home-based, virtual CR has been on rationalizing its utility as well as focusing on the physical activity and exercise component of CR [13]. A systematic review of randomized controlled trials comparing home-based (which may have included digital interventions) with center-based CR determined that home-based CR is noninferior to center-based CR in total mortality, exercise capacity, or health-related quality of life 24 months after CR [14]. Virtual CR has also been endorsed by the American Heart Association for its ability to overcome the accessibility barriers inherent to traditional, in-person, center-based CR [12]. However, how comfortable patients with CVD are with technology, what sociodemographic factors influence their technology use for health care purposes, and whether patients would be interested in using technology to facilitate CR at home have not been fully explored. A cross-sectional study conducted using the Health Information National Trends Survey found that 50% of US adults with CVD reported having a telehealth visit (video or audio‐only) in the past year, with the majority being satisfied with their care [15]. Another cross-sectional study conducted in Israel, involving patients with CVD, found that 83% of the study population was interested in remote digital CR [16]. Younger age, sex, and education level did not impact this interest; however, it is unknown whether this would be the case with prolonged use of virtual CR [12,16].

This study aimed to assess the willingness of patients to engage in virtual CR. Specifically, we aimed to (1) describe patient awareness of, perceived benefits of, and barriers to attending traditional, in-person, center-based CR; (2) characterize technology ownership and digital health use across earlier and later enrollment periods, broadly corresponding to pandemic-era and postacute pandemic care patterns; and (3) evaluate patient interest in virtual CR and identify sociodemographic and clinical factors associated with interest.

## Methods

### Study Design and Participants

In this cross-sectional study with prospective enrollment, we enrolled patients admitted to the progressive cardiac care unit (PCCU) at Johns Hopkins Hospital. Johns Hopkins has an in-person, center-based CR program, but at the time of recruitment for this study, it did not offer virtual CR as part of usual care. Potentially eligible participants were identified based on the following CR-qualifying diagnoses and interventions: acute myocardial infarction (ST-elevation myocardial infarction [STEMI] or non-STEMI [NSTEMI] type 1), angina, heart failure, coronary artery bypass grafting (CABG), coronary artery angioplasty or percutaneous coronary intervention (PCI), heart valve surgery, transcatheter aortic valve replacement (TAVR), or heart transplantation [17]. The inpatient PCCU was selected as the survey setting because hospitalization for a CR-qualifying diagnosis represents a key predischarge opportunity for CR education, referral, and assessment of patient preferences regarding center-based, home-based, and virtual rehabilitation options. Patients were excluded if they declined participation or if the primary team felt they were not appropriate for bedside survey administration because of clinical instability, inability to provide informed consent, cognitive impairment, or another clinical factor that would preclude participation. These decisions were made based on the clinical judgment of the treatment team rather than by a formalized exclusion checklist. Participants were recruited from January 2020 to March 2024. Recruitment was not formally paused during the pandemic; however, in-person recruitment was limited during portions of the earlier study period because of pandemic-related restrictions on bedside research activities and patient contact.

For exploratory temporal analyses of digital health use, participants were grouped into an earlier enrollment period (2020‐2022) and a later enrollment period (2023‐2024). This cutoff was chosen to broadly distinguish patients enrolled during the earlier pandemic-era implementation of telehealth and digital health services from those enrolled after the acute pandemic period and near the end of the COVID-19 public health emergency in 2023 [18]. Because enrollment was imbalanced between these periods, temporal comparisons were considered exploratory. A complete screening log of all CR-eligible patients admitted to the PCCU, patients approached for participation, and patients who declined participation was not maintained throughout the full enrollment period. Therefore, the total number of eligible patients during the study period, the number approached, and the proportion who declined participation could not be reliably determined. Study team members approached patients at the bedside in the PCCU to confirm their eligibility, determine their interest in the study, obtain informed consent, and administer the survey on a tablet or laptop via Qualtrics.

### Ethical Considerations

This study was approved by the Johns Hopkins University School of Medicine Institutional Review Board (IRB00221052), and a waiver of documentation of consent was obtained.

### Measures and Data Collection

A survey developed by the study team and based on the framework of the American Medical Association Digital Health Survey (Multimedia Appendix 1) was administered to the participants in person [19]. The survey comprised multiple-response questions (ie, select all that apply) to collect data on participant demographics (age, sex, race, and educational attainment); technology ownership and past digital health use; knowledge of, attitudes toward, and barriers to attending CR; and attitudes toward virtual CR. In the survey, participants were provided with an explanation of what CR entails, including the length of the program and the various components. The survey was designed for this study and was not formally psychometrically validated. Data were also extracted from participants’ electronic medical records regarding their marital status, employment status, health insurance status, CR-eligible diagnoses and/or interventions, past attendance at CR, and number of hospitalizations in the past 5 years.

### Statistical Analysis

Descriptive statistics were summarized as means with SDs for continuous data with a normal distribution, medians with IQRs for continuous data with a skewed distribution, and frequencies with percentages for categorical data. Chi-square or Fisher exact tests were used to compare technology ownership and digital health use among participants enrolled before or during (2020‐2022) and after (2023‐2024) the COVID-19 pandemic. Given the imbalance in group sample sizes, these temporal comparisons were prespecified as exploratory and were not intended to establish definitive changes attributable to the COVID-19 pandemic [18]. Sociodemographic characteristics of participants (ie, age, sex, race, educational attainment, marital status, employment status, and health insurance status) were presented according to digital health use status, including technology ownership, digital health use, and perceived smartphone benefits to health. Both sociodemographic and clinical characteristics (number of prior hospitalizations in the past 5 years, prior CR attendance, number of qualifying CR diagnoses, and type of qualifying CR diagnosis [medical only, procedural only, or both]) of participants were presented according to CR-related responses by participants including awareness of CR, perceived benefits of CR, barriers to attending CR, and interest in virtual CR. Medical CR-qualifying diagnoses included acute myocardial infarction and/or heart failure, and procedural qualifying CR diagnoses included CABG, PCI, TAVR, and/or heart transplant. Those sociodemographic and clinical characteristics were compared between dichotomized groups of digital health use status and CR-related responses using independent 2-tailed *t* tests or Mann-Whitney *U* tests for continuous data and chi-square or Fisher exact tests for categorical variables.

Complete-case analysis was used for the primary analyses, with denominators reported based on available data for each outcome or covariate. Missing data were primarily due to item-level survey nonresponse and missing electronic medical record–derived variables among early-study participants for whom identifying information was not collected. Because missingness was related in part to early-study data collection procedures, the assumption that data were missing completely at random may not fully hold. Multiple imputation was not performed because the primary outcome, interest in virtual CR, was available for all participants, and missingness primarily affected secondary sociodemographic and clinical subgroup variables. Therefore, subgroup analyses using variables with missing data were interpreted as exploratory.

No formal a priori sample size calculation was performed. The analytic sample represented a consecutive convenience sample of eligible cardiac inpatients who were identified by electronic medical record review, approached by the study team when feasible, and agreed to participate during the enrollment period. No adjustment for multiple comparisons was applied because these subgroup analyses were exploratory and intended to generate hypotheses rather than test a prespecified hierarchy of confirmatory hypotheses. Given the modest sample size and limited number of events in some subgroups, multivariable modeling was not performed for all outcomes because of concern about model overfitting. Therefore, subgroup findings should be interpreted as exploratory and hypothesis generating.

## Results

### Overview

A total of 150 patients were included (mean age 64, SD 13 years; n=57, 38% female; and n=85, 56.7% White). With respect to sociodemographic characteristics, 26% (39/150) of the patients had a high school education or less, 46.7% (70/150) were married, 26.2% (34/130) were employed full time, and 63.8% (83/130) had private insurance (Table 1).

**Table 1.** Baseline characteristics (N=150).

Characteristics	Participants
Sociodemographic characteristics	
Age (y), mean (SD)	64.1 (13.0)
Sex, n (%)	
Male	93 (62)
Female	57 (38)
Race, n (%)	
Asian	6 (4)
Black or African American	51 (34)
White	85 (56.7)
Other	8 (5.3)
Education, n (%)	
High school or less	39 (26)
Greater than high school	111 (74)
Marital status (n=130), n (%)^a^	
Married	70 (53.8)
Unmarried or other	60 (46.2)
Employment status (n=130), n (%)^a^	
Full time or self-employed	34 (26.2)
Other	96 (73.8)
Insurance status (n=130), n (%)^a^	
Private or other	83 (63.8)
Medicare or Medicaid	47 (36.2)
Clinical characteristics	
Number of prior hospitalizations in past 5 years, median (IQR)^a^	3 (2-5)
Prior CR^b^ participation (n=130), n (%)^a^	42 (32.3)
Index hospitalization length of stay (d), median (IQR)^a^	9 (4-16)
Admitted to critical care during index hospitalization (n=130), n (%)^a^	34 (26.2)
Number of CR-qualifying diagnoses or interventions, median (IQR)^a,c^	2 (1-2)
CR-eligible diagnoses or interventions (n=130), n (%)^a^^,c^	
ST-elevation myocardial infarction	23 (17.7)
Non–ST-elevation myocardial infarction type 1	19 (14.6)
Angina	21 (16.2)
Heart failure	85 (65.4)
Coronary artery bypass grafting	26 (20)
Percutaneous coronary intervention	41 (31.5)
Heart valve surgery	20 (15.4)
Transcatheter aortic valve replacement	18 (13.8)
Heart transplant	10 (7.7)
Type of qualifying diagnosis or intervention (n=130), n (%)^a^	
Medical diagnosis only	42 (32.3)
Procedural diagnosis or intervention only	23 (17.7)
Both medical and procedural qualifying diagnoses or interventions	65 (50)

a Denominators vary because of item-level survey nonresponse and missing electronic medical record–derived variables for early-study participants for whom identifying information was not collected. Percentages were calculated among participants with available data for each variable.

b CR: cardiac rehabilitation.

c Participants may have had more than one qualifying CR diagnosis or intervention; therefore, the percentages exceed 100%.

### Interest in Virtual CR

Overall, of 150 participants, 107 (71.3%) expressed interest in virtual CR. Interest in virtual CR was higher among non-White participants than among White participants (55/65, 84.6% vs 52/85, 61.2%; *P*=.002). Interest in virtual CR did not differ significantly by age, sex, education, marital status, employment status, health insurance status, number of prior hospitalizations, number of qualifying diagnoses, type of qualifying diagnosis, or prior CR participation (Table 2). Because multiple subgroup comparisons were performed, these findings should be interpreted as exploratory.

**Table 2.** Sociodemographic and clinical associations with interest in virtual cardiac rehabilitation (CR)^a^.

	No interest in virtual CR	Interest in virtual CR	*P* value^b^
Age (y), mean (SD)	65.7 (13.9)	63.5 (12.7)	.35
Sex, n (%)			.38
Female (n=57)	14 (24.6)	43 (75.4)	
Male (n=93)	29 (31.2)	64 (68.8)	
Race, n (%)			.002
White (n=85)	33 (38.8)	52 (61.2)	
Non-White (n=65)	10 (15.4)	55 (84.6)	
Education, n (%)			.94
High school or less (n=39)	11 (28.2)	28 (71.8)	
Greater than high school (n=111)	32 (28.8)	79 (71.2)	
Marital status, n (%)			.86
Married (n=70)	20 (28.6)	50 (71.4)	
Unmarried (n=60)	18 (30)	42 (70)	
Employment status, n (%)			.37
Full time or self-employed (n=34)	35.3 (12)	64.7 (22)	
Other (n=96)	27.1 (26)	72.9 (70)	
Health insurance, n (%)			.49
Private or other (n=83)	26 (31.3)	57 (68.7)	
Medicare or Medicaid (n=47)	12 (25.5)	35 (74.5)	
Number of prior hospitalizations, median (IQR)	3 (2-5)	3 (2-5)	.89
Sum of qualifying diagnoses, median (IQR)	2 (1-3)	2 (1-2)	.43
Type of qualifying diagnosis, n (%)			.06
Medical diagnosis (n=42)	35 (83.3)	7 (16.7)	
Procedural diagnosis (n=23)	13 (56.5)	10 (43.5)	
Both diagnoses (n=65)	44 (67.7)	21 (32.3)	
Prior CR, n (%)			.26
No prior rehabilitation (n=88)	65 (73.9)	23 (26.1)	
Prior CR (n=42)	27 (64.3)	15 (35.7)	

a Denominators vary because of item-level survey nonresponse and missing electronic medical record–derived variables for early-study participants for whom identifying information was not collected. Percentages were calculated among participants with available data for each variable.

b *P* values were calculated using chi-square or Fisher exact tests for categorical variables, independent *t* tests for normally distributed continuous variables, and Mann-Whitney *U* tests for nonnormally distributed continuous variables, as appropriate.

### Technology Ownership and Digital Health Use

Among the 150 participants, 27 (18%) were enrolled during the earlier enrollment period from 2020 to 2022 and 123 (82%) were enrolled during the later enrollment period from 2023 to 2024. Given this imbalance, comparisons between enrollment periods should be interpreted as exploratory. Smartphone ownership was high in both periods, with 100% (n=27) of participants enrolled in 2020 to 2022 owning a smartphone and 94.3% (115/122) of those enrolled in 2023 to 2024 reporting ownership. The use of smartwatches and wearables was higher in the 2023 to 2024 cohort (40/122, 32.8%) than in the 2020 to 2022 cohort (7/27, 25.9%), although the differences were not statistically significant (*P*=.48).

Some forms of digital health use were higher after the COVID-19 pandemic. Notably, the proportion of participants who texted their health care providers was significantly higher after the pandemic (2020‐2022: 6/27, 22.2%; 2023‐2024: 74/123, 60.7%; *P*<.001). Other forms of digital health use, including health app use (2020‐2022: 11/27, 40.7%; 2023‐2024: 57/123, 49.6%; *P*=.68), were similar, while sharing information from health apps with health care providers (2020‐2022: 25.9%; 2023‐2024: 45.5%; *P*=.08) showed a numerical but nonsignificant increase after the pandemic. Posting health information on social media platforms (2020‐2022: 3/27, 11.1%; 2023‐2024: 6/123, 4.9%; *P*=.37) and watching health-related videos on YouTube (Google Inc; 2020‐2022: 12/27, 44.4%; 2023‐2024: 60/123, 48.8%; *P*=.17) were not significantly different after the pandemic (Table 3).

**Table 3.** Digital health use in participants enrolled before, during, and after the COVID-19 pandemic.

	Enrolled before or during the COVID-19 pandemic (2020‐2022; n=27), n (%)	Enrolled after the COVID-19 pandemic (2023‐2024; n=123), n (%)	*P* value^a^
Health app use	11 (40.7)	57 (46.3)	.68
Shared information from health apps with HCP^b^	7 (25.9)	55 (44.7)	.08
Texted HCP	6 (22.2)	74 (60.2)	<.001
Posted health information on social media	3 (11.1)	6 (4.9)	.37
Watched a health-related video on YouTube	12 (44.4)	60 (48.8)	.17

a *P* values were calculated using chi-square or Fisher exact tests.

b HCP: health care provider.

The proportion of participants who reported that their smartphone assisted in leading a healthier lifestyle was numerically higher after the pandemic (2020‐2022: 29.6%; 2023‐2024: 41.5%; *P*=.15), though this difference did not reach statistical significance. The use of smartphones for learning about illnesses (2020‐2022: 62.3%; 2023‐2024: 75.6%; *P*=.04) and discussing health with health care providers (2020‐2022: 44.4%; 2023‐2024: 69.9%; *P*=.01) were significantly higher after the pandemic (Table 4).

**Table 4.** Perceived smartphone benefits to health in participants enrolled before, during, and after the COVID-19 pandemic (N=150).

	Enrolled before or during the COVID-19 pandemic (2020-2022; n=27), n (%)	Enrolled after the COVID-19 pandemic (2023-2024; n=123), n (%)	*P* value^a^
Smartphone or tablet assisted in living a healthier lifestyle	8 (29.6)	51 (41.5)	.15
Smartphone or tablet assisted in learning about illnesses	17 (63)	93 (75.6)	.04
Smartphone or tablet assisted in talking about health with provider	12 (44.4)	86 (69.9)	.01

a *P* values were calculated using chi-square or Fisher exact tests.

### Predictors of Technology Ownership and Digital Health Use

Smartphone and tablet ownership was consistent across age, sex, race, marital status, education, and employment status. Smartwatch and wearable ownership was also consistent across age, sex, race, marital status, and employment status. However, participants with greater than a high school education had higher rates of smartwatch or wearable device ownership than those with only a high school education or less (41/110, 37.3% vs 6/39, 15.4%; *P*=.01; Multimedia Appendix 2).

White participants were more likely to engage in texting health care providers compared with non-White participants (55/85, 64.7% vs 25/64, 39.1%; *P*=.002). Similarly, participants with greater than a high school education were more likely to use health apps (68/114, 59.4% vs 9/37, 24.3%; *P*=.001) and watch YouTube health videos (59/111, 53.2% vs 13/39, 33.3%; *P*=.03) than participants with a high school education or less. Texting health care providers was numerically higher among participants with greater than a high school education, but this difference was not statistically significant (*P*=.07). Married participants were more likely to use a health app (60.6% vs 33.9%; *P*=.003), share information from health apps with their health care providers (37/70, 52.9% vs 20/58, 34.5%; *P*=.04), and text their health care providers (50/70, 71.4% vs 27/59, 45.8%; *P*=.003) than their unmarried counterparts (Multimedia Appendix 2).

Participants with greater than a high school education were more likely to perceive smartphones as beneficial for leading a healthier lifestyle (50/104, 48.1% vs 9/37, 24.3%; *P*=.01) and learning about illnesses (90/105, 85.7% vs 20/37, 54.1%; *P*<.001) than participants with a high school education or less. Similarly, married participants were more likely to report that smartphones assisted with learning about illnesses (58/66, 87.9% vs 40/56, 71.4%; *P*=.02) and discussing their health with their health care providers (57/66, 86.4% vs 32/55, 58.2%; *P*<.001) than their unmarried counterparts. White participants were more likely to report that smartphones assisted with discussing their health with their health care providers (62/81, 76.5% vs 36/60, 60%; *P*=.04). Individuals employed full time (30/31, 96.8% vs 68/91, 74.7%; *P*=.008) were more likely to report that smartphones assisted with learning about illnesses than their counterparts (Multimedia Appendix 2).

### Awareness of and Barriers to Attending In-Person CR

Awareness of CR (106/149, 71.1%) did not differ by age, sex, race, education, marital status, employment status, number of prior hospitalizations, or number or type of CR-qualifying diagnoses. However, individuals with Medicare or Medicaid insurance were more likely to be aware of CR than individuals with private or other types of insurance (41/47, 87.2% vs 48/82, 58.5%; *P*=.001; Multimedia Appendix 2).

Non-White participants were more likely to report that CR could improve their sense of well-being than White participants (51/57, 89.5% vs 46/62, 74.2%; *P*=.03). Participants not employed full time (74/77, 96.1% vs 21/25, 84%; *P*=.04) were more likely to perceive CR as beneficial for preventing their condition from worsening than their counterparts. Perceived benefit of CR for preventing worsening of disease did not differ significantly by education level. Patients with no prior CR experience were more likely to believe that participating in CR would lower their chances of another cardiac event (69/71, 97.2% vs 26/31, 83.9%; *P*=.01). Individuals with a medical CR-qualifying diagnosis only were more likely to perceive CR as beneficial for preventing their condition from worsening (33/34, 97.1% vs 11/14, 78.6% with a procedural CR-qualifying diagnosis only vs 51/54, 94.4% with both; *P*=.01; Multimedia Appendix 2).

Younger patients (*P*=.001) and males (23/84, 27.4% vs 6/50, 12%; *P*=.04) were more likely to report absence from work as a barrier to attending CR. Full-time employees were more likely to report absence from work (17/33, 51.5% vs 10/87, 11.5%; *P*<.001) and lack of time (16/33, 48.5% vs 24/87, 27.6%; *P*=.03) as barriers to attending CR than their counterparts who were not employed full-time. Full-time employees were less likely to report travel (12/33, 36.4% vs 54/87, 62.1%; *P*=.01) and paperwork (3/33, 9.1% vs 10/87, 11.5%; *P*=.01) as barriers to attending CR than their counterparts who were not employed full-time. Although the difference in paperwork as a barrier reached statistical significance, the absolute difference was small and is unlikely to be clinically meaningful. Patients with a higher number of prior hospitalizations cited travel as a barrier to attending CR (*P*=.02). Individuals with a medical CR-qualifying diagnosis only were more likely to report cost as a barrier to attending CR (19/40, 47.5% vs 5/19, 26.3% with procedural CR-qualifying diagnosis only vs 15/61, 24.6% with both; *P*=.046; Multimedia Appendix 2).

## Discussion

### Principal Results

This cross-sectional study with prospective enrollment explored patient interest in virtual CR by assessing patients’ attitudes toward and barriers to in-person CR, technology ownership, and digital health use. The results showed that while patients understood the benefits of CR, many cited travel, time, absence from work, and paperwork as barriers to attending CR. Barriers related to absence from work and time constraints were noted more frequently among younger patients, men, and full-time employees. Notably, most participants (107/150, 71.3%) expressed interest in virtual CR, especially non-White participants and those with procedural CR-qualifying diagnoses only. The use of digital health tools, including texting health care providers and sharing health data via apps, increased after the COVID-19 pandemic, suggesting a growing readiness among patients to adopt virtual health care solutions.

### Comparison With Prior Work

Our findings are consistent with previous research showing that patients are interested in using virtual CR, especially to address barriers related to travel and time constraints [20]. Multiple studies have already found that home-based CR is as beneficial as center-based CR [14,21,22]. For clarity, center-based CR refers to traditional facility-based programs [10]. Home-based CR involves recovery at home with periodic supervision, and virtual CR is conducted through digital technologies such as telehealth or mobile apps [10]. Additionally, multiple studies have demonstrated that virtual or digital CR may safely facilitate home-based CR and improve adherence by reducing barriers to attendance, such as transportation costs, travel time to CR centers, and capacity constraints of centers [8,13,23,24]. The American Association of Cardiovascular and Pulmonary Rehabilitation, American Heart Association, and American College of Cardiology have stated that home-based CR can reasonably take the place of center-based CR for low- to moderate-risk patients, and digital health interventions can assist in cementing this transition [25].

A prior analysis among persons with atherosclerotic CVD found that health information technology (HIT) use was higher among women, younger adults, White adults, and individuals with a higher income, who were employed, married, and held a bachelor’s degree or higher [26]. HIT use was defined differently in this analysis as the combination of looking up health information on the internet, filling a web-based prescription, scheduling a medical appointment on the internet, communicating with health care providers through email, or using web-based group chats to learn about health topics [26]. Despite differences between the prior NHIS analysis and the present study in how HIT or digital health use was defined, we similarly found that White participants, participants with higher educational attainment, and married participants used digital health tools more frequently.

Notably, non-White participants in our study were more interested in a virtual CR than White participants. Prior studies have described narrowing gaps in digital health use among historically underrepresented racial and ethnic groups, particularly in general telehealth and patient portal use, rather than in virtual CR specifically [27,28]. Our finding extends this broader digital health trend to the CR setting and suggests that virtual CR may be acceptable to patient groups that have historically faced barriers to traditional center-based CR. Further research is needed to determine whether this interest translates into virtual CR enrollment, adherence, and completion and to identify implementation strategies that support equitable access.

Nevertheless, these findings highlight the importance of ensuring that virtual CR platforms are designed to be accessible to all patients, regardless of race, age, gender, income level, or digital literacy. Barriers to CR are an important consideration for its consistent use in patient populations. The usability and reliability of virtual CR interfaces, the support systems patients have access to when needing help with technology, the consideration of frailty in older adult patients in a nonclinical setting, and the lasting psychosocial effects of undergoing an adverse cardiac event are all potential challenges that must be addressed before virtual CR can be fully accepted by patients and physicians [12,29-32].

Our findings suggest that many cardiac inpatients are receptive to virtual CR, particularly in the context of commonly reported barriers to traditional, in-person, center-based CR. Rather than replacing center-based CR, virtual and hybrid CR models may serve as complementary approaches that expand access for patients who face barriers related to travel, time, work obligations, or other logistical constraints. These findings support further evaluation of virtual CR implementation, usability, adherence, and clinical outcomes in larger, multicenter studies. Importantly, efforts to expand virtual CR should also address digital equity, including access to devices, internet connectivity, digital literacy, language accessibility, and technical support.

### Strengths and Limitations

This study has several strengths, including its timing before, during, and after the COVID-19 pandemic, which allowed us to capture patient digital health use during different periods. Additionally, our focus on a diverse population with a range of CR-qualifying cardiac conditions provides valuable insights into how different patient subgroups may approach virtual CR.

Limitations include the relatively small sample size and the conduct of the study at a single academic medical center, limiting our ability to conduct multivariate modeling as well as the generalizability of the findings. In addition, the manuscript includes multiple subgroup comparisons without statistical correction for multiple testing, which increases the risk of type I error. These analyses were exploratory and should be interpreted as hypothesis generating rather than confirmatory.

The enrollment-period comparison was also limited by substantial imbalance in group size, with 27 participants enrolled in 2020‐2022 and 123 participants enrolled in 2023‐2024. This imbalance limited statistical power for temporal comparisons and increased the risk that observed differences reflect recruitment patterns, pandemic-related disruptions, or secular trends in digital health adoption rather than true differences attributable to the COVID-19 pandemic. Therefore, these comparisons should be interpreted as exploratory and not as definitive evidence of pandemic-related changes.

In addition, because a complete screening log was not maintained, we could not determine how many eligible patients were admitted during the study period, how many were approached, or how many declined participation. This limits our ability to assess the recruitment rate and nonresponse bias and raises the possibility of selection bias, particularly if patients who were more clinically stable, more comfortable with technology, or more interested in CR were more likely to participate.

The survey instrument was developed for this study and was not formally psychometrically validated. Therefore, responses may be subject to measurement error, and self-reported interest in virtual CR may not fully predict actual postdischarge enrollment, adherence, or completion. Because surveys were administered at the bedside by study team members, responses may have been subject to social desirability bias, potentially inflating reported interest in virtual CR or perceived benefits of CR. Future studies should consider anonymous or self-administered survey formats to reduce this potential bias. Digital health attitudes during the study period may also have been influenced by pandemic-driven adaptation to telehealth and remote care rather than reflecting stable long-term preferences. Because this study assessed attitudes at a single time point during hospitalization, reported interest in virtual CR may not translate into actual postdischarge engagement, enrollment, adherence, or completion. Future longitudinal studies should assess whether inpatient interest in virtual CR persists after discharge and should evaluate CR enrollment, participation, and completion at clinically relevant follow-up points, including 6 and 12 months.

Another limitation is that identifying information was not collected for respondents enrolled at the beginning of the study; consequently, we were unable to collect complete sociodemographic and clinical characteristics from the electronic health record for these individuals, resulting in approximately 13% missing data for certain variables. Additionally, since the population surveyed comprised patients on the PCCU, the sample may not be representative of all cardiac patients admitted to general medicine floors or other locations. Additionally, participants were enrolled at one academic medical center with strong CR infrastructure, which may not reflect practices at other institutions. Because enrollment was limited to English-speaking patients, our findings may not be transferable to patients with limited English proficiency or to linguistically diverse populations. This limitation is particularly relevant to the equity implications of virtual CR, as language access may influence digital health use, patient education, and engagement with rehabilitation programs. Moreover, race was analyzed as White versus non-White, which aggregates heterogeneous racial and ethnic groups and may obscure important differences in digital health use, barriers to CR, and interest in virtual CR across specific populations. Future studies with larger and more diverse samples should disaggregate racial and ethnic categories to better understand subgroup-specific barriers, preferences, and implementation needs.

Finally, while we captured a range of sociodemographic and clinical characteristics, other factors such as health literacy, digital literacy, quality of internet access, data plan limitations, comfort with troubleshooting technology, and access to caregiver or technical support were not captured. Therefore, smartphone ownership should not be interpreted as equivalent to digital readiness, and our findings may overestimate patients’ ability to successfully engage with virtual CR without additional support.

### Conclusions

Overall, our findings suggest that patients with CR-eligible diagnoses face substantial barriers to attending in-person CR but are interested in using virtual CR to overcome logistical barriers. Given the single-site design and modest sample size, further research is needed to confirm the generalizability of these findings across care settings. Investigating why non-White patients showed greater interest in virtual CR is an important direction for future research.

## Supplementary material

10.2196/91767 Multimedia Appendix 1 Patient survey.

10.2196/91767 Multimedia Appendix 2 Supplemental exploratory subgroup analyses of technology ownership, digital health use, perceived smartphone benefits, cardiac rehabilitation awareness, perceived cardiac rehabilitation benefits, and barriers to attending cardiac rehabilitation.

## Abbreviations

CABG: coronary artery bypass grafting
CR: cardiac rehabilitation
CVD: cardiovascular disease
HIT: health information technology
NSTEMI: non–ST-elevation myocardial infarction
PCCU: progressive cardiac care unit
PCI: percutaneous coronary intervention
STEMI: ST-elevation myocardial infarction
TAVR: transcatheter aortic valve replacement
