# Supplementary material for: Digital Health Use and Interest in Virtual Cardiac Rehabilitation Among Cardiac Inpatients: Cross-Sectional Study
Source: JMIR Cardio. 2026 Sep 11;10:e91767. doi: 10.2196/91767 (PMC13566072; doi:10.2196/91767)
Supplement: Supplementary file 1 [file cardio-v10-e91767-s001.docx]

Cardiac Mega Survey Patient Facing Questions - V1

Start of Block: Block 5

Q51 **Assessing Interest and Utilization of Mobile Health (mHealth) Applications Among Cardiac Inpatients** 
Purpose: The purpose of this research study is to better understand how patients use mobile health applications or other digital health tools, if they do at all. This study is administered through a survey, which has 4 major parts. This study is expected to take 15-20 minutes.   
 
We will collect information about you in this study, using your responses from this survey and your medical record to look at  your past medical history, including cardiac diagnoses and medications. People at Johns Hopkins who are involved in the study or who need to make sure the study is being done correctly will see the information. People at Johns Hopkins may need to send your information to people outside of Johns Hopkins (for example, government groups like the Food and Drug Administration) who need to make sure the study is being done correctly.   
     We will continue to collect information about you until the end of the study unless you tell us that you have changed your mind. If you change your mind and do not want your information to be used for the study, you must contact the Principal Investigator by using the contact information provided in this document.  Your cancellation will not affect information already collected in the study, or information that has already been shared with others before you cancelled your authorization.   We try to make sure that everyone who needs to see your information uses it only for the study and keeps it confidential - but, we cannot guarantee this.     
We do not anticipate any risks or discomfort from participating in this study. Similarly, we do not anticipate direct benefit to you from taking the study, but participation in this study may provide you with information about digital health technology to further discuss with your clinician.   
 
Please check off all the boxes below to continue with the survey. Continuation of this survey will act as informed consent.

- I am at least 18 years of age. (1)
- I understand the purpose of the study. I understand that refusal to participate has no impact on my medical care, and that I can stop at any point in time. (2)
- I consent to participate in this study. (3)

End of Block: Block 5

Start of Block: Participant Demographic Information

AnonID To protect your privacy, we will create an anonymous ID for you. Please type out the last 2 letters of your first name + the date of your birthday  + last 3 numbers of your zip code.

*Example: Jane Doe is born on January 5th. Her zip code is 21205. Her Anonymous ID would be NE05205.*

________________________________________________________________

Age Age:

▼ 18 (1) ... 110 (93)

Sex Sex:

- Male (1)
- Female (2)
- Decline to state (3)

Q4 Race:

- White (1)
- Black or African American (2)
- American Indian or Alaska Native (3)
- Asian (4)
- Native Hawaiian or Pacific Islander (5)
- Other (6)

Q5 Hispanic, Latino, or Spanish origin:

- Yes (1)
- No (2)

Q6 Highest level of education

- Less than high school (1)
- High school graduate (2)
- Some college (3)
- 2 year degree (4)
- 4 year degree (5)
- Professional degree (6)
- Doctorate (7)

End of Block: Participant Demographic Information

Start of Block: Baseline Knowledge of Admission

Q7 Why are you in the hospital? Please share your diagnosis, if you know it.

________________________________________________________________

End of Block: Baseline Knowledge of Admission

Start of Block: Baseline Digital Health Information

Q9 Do you own any of the following? Circle/check all that apply.

- Smartphone (1)
- Electronic tablet, like Kindle Fire or iPad (2)
- Smartwatch (3)
- FitBit (4)
- Blood pressure monitor (5)
- Blood glucose (sugar) monitor (6)

Display This Question:

If Do you own any of the following? Circle/check all that apply. = Smartphone

Q52 Do you own an iPhone or Android smartphone?

- iPhone (1)
- Android (2)

Display This Question:

If Do you own any of the following? Circle/check all that apply. = Smartwatch

Q53 What type of smartwatch do you own?

- Apple Watch (1)
- Other smartwatch device (2)

Display This Question:

If Do you own any of the following? Circle/check all that apply. = Smartphone

Or Do you own any of the following? Circle/check all that apply. = Electronic tablet, like Kindle Fire or iPad

Or Do you own any of the following? Circle/check all that apply. = Smartwatch

Q10 Do you have any health apps on your smartphone or tablet?

- Yes (please list): (1) __________________________________________________
- No (2)

Q11 In the past year, have you:

|  | Yes (1) | No (2) |
| --- | --- | --- |
| shared information from your health apps or device with your healthcare provider? (1) |  |  |
| texted your healthcare provider? (2) |  |  |
| posted health information on social media? (3) |  |  |
| watched a health-related video on YouTube? (4) |  |  |

Display This Question:

If Do you own any of the following? Circle/check all that apply. = Smartphone

Or Do you own any of the following? Circle/check all that apply. = Electronic tablet, like Kindle Fire or iPad

Or Do you own any of the following? Circle/check all that apply. = Smartwatch

Q12 Has your tablet or smartphone helped you:

|  | Yes (1) | No (2) |
| --- | --- | --- |
| live a healthier life like quit smoking, lose weight, or exercise more? (4) |  |  |
| learn about an illness? (5) |  |  |
| talk about your health with your provider? (6) |  |  |

End of Block: Baseline Digital Health Information

Start of Block: Cardiac Rehab Questions

Q13 Have you ever heard of Cardiac Rehabilitation ("cardiac rehab")?

- Yes (1)
- No (2)

Display This Question:

If Have you ever heard of Cardiac Rehabilitation ("cardiac rehab")? = Yes

Q14 What do you think are the main goals of Cardiac Rehabilitation Programs?

________________________________________________________________

Q17 "Cardiac Rehabilitation" or ‘’Cardiac Rehab" is a structured program for people with heart problems. In Cardiac Rehab, specialized staff teach people how to keep their heart healthy. Patients doing Cardiac Rehab live longer, have fewer heart attacks and stay away from the hospital more. Patients enrolled in the program visit a specialized Cardiac Rehab center around 3 times a week for 12 weeks. During these sessions, the medical team works with the patients and makes sure the program fits their needs. The components of Cardiac Rehab are shown below. The Cardiac Rehab team teaches patients how to exercise. Patients learn which foods are healthiest and which ones to avoid. Patients learn ways to manage their stress and get support for any feelings of sadness or worry. Patients learn about cardiovascular disease and get help managing their medications. If patients smoke, they get help with quitting smoking. 
 

 *Horwich TB, Fonarow GC. Should I Participate in a Cardiac Rehabilitation Program?. JAMA cardiology. 2018 Nov 1;3(11):1136-.*

Q18  Do you think that you could benefit from Cardiac Rehabilitation?

- Yes (1)
- No (3)

Display This Question:

If  Do you think that you could benefit from Cardiac Rehabilitation? = Yes

Q19 How would you expect to benefit? I would like to: (Check all that apply)

- lower the chances of another cardiac event. (1)
- prevent my condition from getting worse. (2)
- improve my ability to perform everyday activities and physical exercise. (3)
- improve my sense of well-being (lose weight, quit smoking). (4)
- understand my condition and learn ways to manage it more effectively. (5)
- Other. Please explain. (6) __________________________________________________

Display This Question:

If  Do you think that you could benefit from Cardiac Rehabilitation? = No

Q54 Please explain why. Check all that apply.

- I am happy with my health now. (1)
- I feel that I am too sick to benefit. (2)
- I do not think that Cardiac Rehabilitation works. (3)
- Physical exercise is not safe for me. (4)
- Other. Please explain. (5) __________________________________________________

Q20 What would make it hard for you to participate in a Cardiac Rehabilitation Program, even if you wanted to? Check all that apply.

- Costs/Insurance coverage (1)
- Traveling to the Cardiac Rehab Center (long distance from home, means of transportation, parking) (2)
- Absence from work (3)
- Paperwork (4)
- Time/Tight schedule (5)
- Other (6) __________________________________________________

Q21 Would you be interested in participating in a Cardiac Rehab program delivered from home using digital technology? * This question is solely hypothetical and meant to gauge interest. If you answer "yes", you will not be enrolled in a virtual Cardiac Rehab program.

- Yes (1)
- No (2)

Q22 Would you like to start this program as soon as you leave the hospital?

- Yes (1)
- No (2)

Q23 Do you think that a daily routine cardiac rehab program that is tailored for your needs would be better for you?

- Yes (1)
- No (2)

Q24 Do you think that taking part in a cardiac rehab program that offers you constant feedback on your progress would motivate you to do your best?

- Yes (1)
- No (2)

Q25 Would you be interested in a cardiac rehab program that you could follow from your home for an extended period (longer than most center-based programs)?

- Yes (1)
- No (2)

Q26 Would you feel less safe training on your own rather than in a supervised center?

- Yes (1)
- No (2)

Display This Question:

If Would you feel less safe training on your own rather than in a supervised center? = Yes

Q27 Would the ability of directly communicating through your phone with specialized staff during your training sessions make you feel more confident?

- Yes (1)
- No (2)

Q28 Would you feel less motivated training on your own instead of in a center with other participants?

- Yes (1)
- No (2)

Display This Question:

If Would you feel less motivated training on your own instead of in a center with other participants? = Yes

Q29 Would a group video call with other participants during your training sessions get you more engaged?

- Yes (1)
- No (2)

Q55  If you could design a Cardiac Rehab program on your own, how would you design it so that it would be ideal for you? Please try to imagine how you would like to be treated from your first day in the hospital before doing Cardiac Rehab, while doing Cardiac Rehab, and after Cardiac Rehab.(open-ended)

________________________________________________________________

End of Block: Cardiac Rehab Questions

Start of Block: Corrie App Questions

Q56


 
The following questions will be about Corrie, a health app created by a team of Hopkins cardiologists, nurses, and engineers in collaboration with Apple. Corrie was originally designed for patients who have a heart attack, but offers features that can be helpful for patients with different heart problems.
 
We would like to ask you for your thoughts about the features from Corrie, and whether you would find it helpful to manage your heart health needs. Please watch this brief video to learn a bit more about Corrie.

Q30 How would you like to sign into Corrie?

- Password (1)
- Swipe pattern (2)
- Fingerprint (3)
- Facial recognition (4)

Q31 How helpful would you find each of the following in getting started?

|  | Extremely helpful (1) | Slightly helpful (2) | Not at all helpful (3) |
| --- | --- | --- | --- |
| Corrie "Intro" Video (1) |  |  |  |
| CorrieKit Handout (paper instructions) (2) |  |  |  |

Q32 If we were to create a video teaching you how to use Corrie, what information would you like it to have? Check/circle all that apply.

- How to download the Corrie app (1)
- How to pair the Corrie app with the Apple Watch and blood pressure cuff (2)
- How to enter medications into the Corrie app (3)
- Other (4) __________________________________________________

Q33 How helpful would each of the following features be in helping you take your medications?

|  | Extremely helpful (1) | Slightly helpful (2) | Not at all helpful (3) |
| --- | --- | --- | --- |
| Being able to check off your medications after you take them (1) |  |  |  |
| Having alerts that tell you it’s time to take your medication (2) |  |  |  |
| Have a progress bar to see how good you are at taking medications on time (3) |  |  |  |

Q34 Do you think the following information would help you take a medication safely?

|  | Yes (1) | No (2) |
| --- | --- | --- |
| Adherence (1) |  |  |
| Dosage (2) |  |  |
| Dose times (3) |  |  |
| What the medication does (4) |  |  |
| Medication side effects (5) |  |  |

Q35 Do you think you would be able to add all your medications into Corrie by yourself?

- Yes (1)
- No (2)

Q36 After watching the video on how to use a blood pressure monitor, is this something you could do at home with Corrie?

- Yes (1)
- Maybe (2)
- No (3)

Q37 What would you measure once a day on Corrie after leaving the hospital? Check all that apply.

- Heart rate (1)
- Blood pressure (2)
- Mood (3)
- Weight (4)

Q38 Would it be useful to have a smartwatch that:

|  | Yes (1) | No (2) |
| --- | --- | --- |
| connects to Corrie to automatically track your heart rate and steps? (1) |  |  |
| reminds you about your doctor appointments and medications? (2) |  |  |

Q39 Do you think the videos and articles within the Corrie app would help you learn about your diagnosis and care plan while in the hospital?

|  | Yes (1) | No (2) |
| --- | --- | --- |
| Videos (1) |  |  |
| Articles (2) |  |  |

Q40  Would you call to make a doctor's appointment while in the hospital if the doctor's number and office location were given to you?

- Yes (1)
- Maybe (2)
- No (3)

Q41 Would a tracking system help you schedule follow-up appointments?

- Yes (1)
- No (2)

Q42 Would it help if your appointments were automatically added to your calendar?

- Yes (1)
- No (2)

Q43 Would the “TO-DOs FOR NEXT APPOINTMENT” feature help you keep notes related to your appointment?

- Yes (1)
- No (2)

Q44 Would it be helpful to have the hospital Care Team photos, names, and phone numbers available to you in a place like MyConnect in the Corrie app? * MyConnect is tab in the Corrie app where you can store information, like names and phone numbers, about your care team, family, and friends.

- Yes (1)
- No (2)

Q45 Would it be helpful to have a place to keep your friends, family, and providers all in one place like MyConnect in the Corrie app?

- Yes (1)
- No (2)

Q46 There is an option to securely store images of your insurance cards, stent cards, and photos. Would you find this helpful?

- Yes (1)
- No (2)

Q47 After seeing all the features, would you want to have Corrie available to you in the hospital and at home?

- Yes (1)
- No (2)

Q48 Would you find it easier to learn about your diagnosis and care plan using Corrie compared to paper instructions?

- Yes (1)
- No (2)
- I don't know (3)

Q49 Corrie uses the latest in data security to protect patients’ health information. If you were using Corrie, would you allow your data to be used in research to help us learn more about how to improve heart health?

- Yes (1)
- No (2)

Q50 Do you think Corrie could help you reach your health goals?

- Yes (1)
- No (2)

End of Block: Corrie App Questions
