# Supplementary material for: Digital Health Use and Interest in Virtual Cardiac Rehabilitation Among Cardiac Inpatients: Cross-Sectional Study
Source: JMIR Cardio. 2026 Sep 11;10:e91767. doi: 10.2196/91767 (PMC13566072; doi:10.2196/91767)
Supplement: Supplementary file 2 [file cardio-v10-e91767-s002.docx]

**Appendix 2. Supplemental Exploratory Subgroup Analyses**

The following supplemental tables present exploratory subgroup analyses of technology ownership, digital health use, perceived smartphone benefits, cardiac rehabilitation awareness, perceived cardiac rehabilitation benefits, and barriers to attending cardiac rehabilitation.

**Table 1. Sociodemographic Associations With Technology Ownership Among Cardiac Inpatients (N=150)**

|  | | **All, N=150** | **Smartphone** | | | **Smartwatch/Wearable** | | |
| --- | --- | --- | --- | --- | --- | --- | --- | --- |
|  |  |  | **No**  **(n=7)** | **Yes**  **(n=142)** | **P-value^a^** | **No**  **(n=102)** | **Yes**  **(n=47)** | **P-value^a^** |
| **Age, yr^b^** | | **64.1 (13.0)** | **72.3 (10.0)** | **63.7 (13.1)** | **0.07** | **63.6 (12.8)** | **65.1 (13.6)** | **0.51** |
| **Sex** | |  |  |  |  |  |  |  |
|  | Women | 38.0 (57) | 5.3 (3) | 94.7 (54) | 0.80 | 63.2 (36) | 36.8 (21) | 0.27 |
|  | Men | 62.0 (93) | 4.3 (4) | 95.7 (88) |  | 71.7 (66) | 28.3 (26) |  |
| **Race** | |  |  |  |  |  |  |  |
|  | White | 56.7 (85) | 3.6 (3) | 96.4 (81) | 0.46 | 67.9 (57) | 32.1 (27) | 0.86 |
|  | Non-white | 43.3 (65) | 6.1 (4) | 93.9 (61) |  | 69.2 (45) | 30.8 (20) |  |
| **Education** | |  |  |  |  |  |  |  |
|  | High school or less | 26 (39) | 5.1 (2) | 94.9 (37) | 0.88 | 84.6 (33) | 15.4 (6) | **0.01^d^** |
|  | Greater than high school | 74 (111) | 4.5 (5) | 95.5 (105) |  | 62.7 (69) | 37.3 (41) |  |
| **Marital status^c^** | |  |  |  |  |  |  |  |
|  | Married | 46.7 (70) | 4.4 (3) | 95.7 (66) | 0.56 | 59.4 (41) | 40.6 (28) | 0.06 |
|  | Unmarried | 40.0 (60) | 6.7 (4) | 93.3 (56) |  | 75.0 (45) | 25.0 (15) |  |
| **Employment status^c^** | |  |  |  |  |  |  |  |
|  | Full-time/self-employed | 26.2 (34) | 6.1 (2) | 93.9 (31) | 0.85 | 69.7 (23) | 30.3 (10) | 0.67 |
|  | Other | 73.9 (96) | 5.2 (5) | 94.8 (91) |  | 65.6 (63) | 34.4 (33) |  |
| **Health insurance^c^** | |  |  |  |  |  |  |  |
|  | Private/other | 63.4 (83) | 6.1 (5) | 93.9 (77) | 0.66 | 67.1 (55) | 32.9 (27) | 0.90 |
|  | Medicare/ Medicaid | 36.2 (47) | 4.3(2) | 95.7 (45) |  | 66.0 (31) | 34.0 (16) |  |

^a^ P values were calculated using chi-square or Fisher exact tests for categorical variables, independent t tests for normally distributed continuous variables, and Mann-Whitney U tests for nonnormally distributed continuous variables, as appropriate.

^b^ Continuous variables are presented as mean (SD), and categorical variables are presented as % (n), unless otherwise indicated.

^c^ Denominators vary because of item-level survey nonresponse and missing electronic medical record-derived variables for early-study participants for whom identifying information was not collected. Percentages were calculated among participants with available data for each variable.

^d^ P<.05.

**Table 2. Sociodemographic Associations With Digital Health Use Among Cardiac Inpatients**

|  | | **Health App Use**  **(N=142)** | | | | **Information Sharing with HCP (N=148)** | | | **Texting HCP**  **(N=149)** | | | **Watching YouTube Health Videos (N=150)** | | |
| --- | --- | --- | --- | --- | --- | --- | --- | --- | --- | --- | --- | --- | --- | --- |
|  |  | **No (n=74)** | **Yes (n=68)** | | **P-value^a^** | **No**  **(n=86)** | **Yes (n=62)** | **P- value^a^** | **No**  **(n=69)** | **Yes (n=80)** | **P-value^a^** | **No**  **(n=78)** | **Yes**  **(n=72)** | **P-value^a^** |
| **Age, yr^b^** | | **64.0 (12.6)** | **63.4 (13.6)** | | **0.78** | **63.5 (12.5)** | **64.8 (13.9)** | **0.54** | **63.1 (13.4)** | **65.0 (12.8)** | **0.38** | **65.8 (11.8)** | **62.3 (14.1)** | **0.09** |
| **Sex** | |  | |  |  |  |  |  |  |  |  |  |  |  |
|  | Women | 53.7 (29) | 46.3 (25) | | 0.77 | 55.4 (31) | 44.6 (25) | 0.60 | 42.1 (24) | 57.9 (33) | 0.42 | 54.4 (31) | 45.6 (26) | 0.65 |
|  | Men | 51.1 (45) | 48.9 (43) | |  | 59.8 (55) | 40.2 (37) |  | 48.9 (45) | 51.1 (47) |  | 50.5 (47) | 49.5 (46) |  |
| **Race** | |  | |  |  |  |  |  |  |  |  |  |  |  |
|  | White | 47.6 (39) | 52.4 (42) | | 0.28 | 54.1 (46) | 45.9 (39) | 0.25 | 35.3 (30) | 64.7 (55) | **0.002^d^** | 48.2 (41) | 51.8 (44) | 0.29 |
|  | Non-white | 57.4 (35) | 42.6 (26) | |  | 63.5 (40) | 36.5 (23) |  | 60.9 (39) | 39.1 (25) |  | 56.9 (37) | 43.1 (28) |  |
| **Education** | |  | |  |  |  |  |  |  |  |  |  |  |  |
|  | High school or less | 75.7 (28) | 24.3 (9) | | **0.001^d^** | 59.0 (23) | 41.0 (16) | 0.90 | 59.0 (23) | 41.0 (16) | 0.07 | 66.7 (26) | 33.3 (13) | **0.03^d^** |
|  | Greater than high school | 40.6 (46) | 59.4 (68) | |  | 57.8 (63) | 42.2 (46) |  | 41.8 (46) | 58.2 (64) |  | 46.8 (52) | 53.2 (59) |  |
| **Marital status^c^** | |  | |  |  |  |  |  |  |  |  |  |  |  |
|  | Married | 39.4 (26) | 60.6 (40) | | **0.003^d^** | 47.1 (33) | 52.9 (37) | **0.04^d^** | 28.6 (20) | 71.4 (50) | **0.003^d^** | 47.1 (33) | 52.9 (37) | 0.28 |
|  | Unmarried | 66.1 (37) | 33.9 (19) | |  | 65.5 (38) | 34.5 (20) |  | 54.2 (32) | 45.8 (27) |  | 56.7 (34) | 43.3 (26) |  |
| **Employment status^c^** | |  | |  |  |  |  |  |  |  |  |  |  |  |
|  | Full-time/self-employed | 41.9 (13) | 58.1 (18) | | 0.21 | 57.6 (19) | 42.4 (14) | 0.78 | 41.2 (14) | 58.8 (20) | 0.90 | 44.1 (15) | 55.9 (19) | 0.31 |
|  | Other | 55.0 (50) | 45.0 (41) | |  | 54.7 (52) | 45.3 (43) |  | 40.0 (38) | 60.0 (57) |  | 53.0 (52) | 47.0 (44) |  |
| **Health insurance^c^** | |  | |  |  |  |  |  |  |  |  |  |  |  |
|  | Private/ other | 53.2 (41) | 46.8 (36) | | 0.64 | 61.7 (50) | 38.3 (31) | 0.06 | 47.6 (39) | 52.4 (43) | **0.03^d^** | 47.0 (39) | 53.0 (44) | 0.17 |
|  | Medicare/ Medicaid | 48.9 (22) | 51.1 (23) | |  | 44.7 (21) | 55.3 (26) |  | 27.7 (13) | 72.3 (34) |  | 59.6 (28) | 40.4 (19) |  |

^a^ P values were calculated using chi-square or Fisher exact tests for categorical variables, independent t tests for normally distributed continuous variables, and Mann-Whitney U tests for nonnormally distributed continuous variables, as appropriate.

^b^ Continuous variables are presented as mean (SD), and categorical variables are presented as % (n), unless otherwise indicated.

^c^ Denominators vary because of item-level survey nonresponse and missing electronic medical record-derived variables for early-study participants for whom identifying information was not collected. Percentages were calculated among participants with available data for each variable.

^d^ P<.05.

**Table 3. Sociodemographic Associations With Perceived Smartphone Benefits to Health**

|  | | **Assisted with Healthier Lifestyle (N=141)** | | | **Assisted with Learning about an Illness (N=142)** | | | **Assisted with Discussing Health with Provider (N=141)** | | |
| --- | --- | --- | --- | --- | --- | --- | --- | --- | --- | --- |
|  |  | **No**  **(n=82)** | **Yes**  **(n=59)** | **P-value^a^** | **No**  **(n=32)** | **Yes**  **(n=110)** | **P-value^a^** | **No**  **(n=43)** | **Yes**  **(n=98)** | **P-value^a^** |
| **Age, yr^b^** | | **64.7 (11.6)** | **62.2 (15.0)** | **0.27** | **66.5 (12.8)** | **62.9 (13.1)** | **0.17** | **62.1 (13.0)** | **64.4 (13.2)** | **0.35** |
| **Sex** | |  |  |  |  |  |  |  |  |  |
|  | Women | 63.0 (34) | 37.0 (20) | 0.36 | 22.2 (12) | 77.8 (42) | 0.94 | 37.0 (20) | 63.0 (34) | 0.18 |
|  | Men | 55.2 (48) | 44.8 (39) |  | 22.7 (20) | 77.3 (68) |  | 23.0 (23) | 77.0 (64) |  |
| **Race** | |  |  |  |  |  |  |  |  |  |
|  | White | 60.5 (49) | 39.5 (32) | 0.51 | 17.3 (14) | 82.7 (67) | 0.08 | 23.5 (19) | 76.5 (62) | **0.04^d^** |
|  | Non-white | 55.0 (33) | 45.0 (27) |  | 29.5 (18) | 70.5 (43) |  | 40.0 (24) | 60.0 (36) |  |
| **Education** | |  |  |  |  |  |  |  |  |  |
|  | High school or less | 75.7 (28) | 24.3 (9) | **0.01^d^** | 45.9 (17) | 54.1 (20) | **<0.001^d^** | 40.5 (15) | 59.5 (22) | 0.12 |
|  | Greater than high school | 51.9 (54) | 48.1 (50) |  | 14.3 (15) | 85.7 (90) |  | 26.9 (28) | 73.1 (76) |  |
| **Marital status^c^** | |  |  |  |  |  |  |  |  |  |
|  | Married | 62.1 (41) | 37.9 (25) | 0.30 | 12.1 (8) | 87.9 (58) | **0.02^d^** | 13.6 (9) | 86.4 (57) | **<0.001^d^** |
|  | Unmarried | 52.7 (29) | 47.3 (26) |  | 28.6 (16) | 71.4 (40) |  | 41.8 (23) | 58.2 (32) |  |
| **Employment status^c^** | |  |  |  |  |  |  |  |  |  |
|  | Full-time/self-employed | 27.5 (14) | 72.6 (17) | 0.10 | 3.2 (1) | 96.8 (30) | **0.008^d^** | 22.6 (7) | 77.4 (24) | 0.57 |
|  | Other | 62.2 (56) | 37.8 (34) |  | 25.3 (23) | 74.7 (68) |  | 27.8 (25) | 72.2 (65) |  |
| **Health insurance^c^** | |  |  |  |  |  |  |  |  |  |
|  | Private/other | 57.9 (44) | 42.1 (32) | 0.99 | 22.1 (17) | 77.9 (60) | 0.38 | 27.6 (21) | 72.4 (55) | 0.70 |
|  | Medicare/Medicaid | 57.8 (26) | 42.2 (19) |  | 15.6 (7) | 84.4 (38) |  | 24.4 (11) | 75.6 (34) |  |

^a^ P values were calculated using chi-square or Fisher exact tests for categorical variables, independent t tests for normally distributed continuous variables, and Mann-Whitney U tests for nonnormally distributed continuous variables, as appropriate.

^b^ Continuous variables are presented as mean (SD), and categorical variables are presented as % (n), unless otherwise indicated.

^c^ Denominators vary because of item-level survey nonresponse and missing electronic medical record-derived variables for early-study participants for whom identifying information was not collected. Percentages were calculated among participants with available data for each variable.

^d^ P<.05.

**Table 4. Sociodemographic and Clinical Associations With Awareness of Cardiac Rehabilitation (N=149)**

|  | | **All, N=149** | **Awareness of Cardiac Rehab** | | |
| --- | --- | --- | --- | --- | --- |
|  |  |  | **No**  **(n=43)** | **Yes**  **(n=106)** | **P-value^a^** |
| **Age, yr^b^** | | **64.2 (13)** | **63.8 (13.1)** | **64.4 (13.0)** | **0.80** |
| **Sex** | |  |  |  |  |
|  | Women | 37.6 (56) | 23.2 (13) | 76.8 (43) | 0.24 |
|  | Men | 62.4 (93) | 32.3 (30) | 67.7 (63) |  |
| **Race** | |  |  |  |  |
|  | White | 57.1 (85) | 25.9 (22) | 74.1 (63) | 0.36 |
|  | Non-White | 43.0 (64) | 32.8 (21) | 67.2 (43) |  |
| **Education** | |  |  |  |  |
|  | High school or less | 25.5 (38) | 39.5 (15) | 60.5 (23) | 0.09 |
|  | Greater than high school | 74.5 (111) | 25.2 (28) | 74.8 (83) |  |
| **Marital status^c^** | |  |  |  |  |
|  | Married | 54.3 (70) | 25.7 (18) | 74.3 (52) | 0.16 |
|  | Unmarried | 45.7 (59) | 37.3 (22) | 62.7 (37) |  |
| **Employment status^c^** | |  |  |  |  |
|  | Full-time/self-employed | 26.4 (34) | 32.4 (11) | 67.6 (23) | 0.84 |
|  | Other | 73.6 (95) | 30.5 (29) | 69.5 (66) |  |
| **Health insurance^c^** | |  |  |  |  |
|  | Private/other | 63.6 (82) | 41.5 (34) | 58.5 (48) | **0.001^d^** |
|  | Medicare/Medicaid | 36.4 (47) | 12.8 (6) | 87.2 (41) |  |
| Number of prior hospitalizations^c^ | | 3 (2-5)) | 3 (2-5) | 3 (2-5) | 1.00 |
| Sum of qualifying diagnoses^c^ | | 2 (1-2) | 2 (1-2) | 2 (1-3) | 0.26 |
| **Type of qualifying diagnosis^c^** | |  |  |  |  |
|  | Medical diagnosis | 31.8 (41) | 41.5 (17) | 58.5 (24) | 0.19 |
|  | Procedural diagnosis | 17.8 (23) | 21.7 (5) | 78.3 (18) |  |
|  | Both diagnoses | 50.4 (65) | 27.7 (18) | 72.3 (47) |  |

^a^ P values were calculated using chi-square or Fisher exact tests for categorical variables, independent t tests for normally distributed continuous variables, and Mann-Whitney U tests for nonnormally distributed continuous variables, as appropriate.

^b^ Continuous variables are presented as mean (SD), and categorical variables are presented as % (n), unless otherwise indicated.

^c^ Denominators vary because of item-level survey nonresponse and missing electronic medical record-derived variables for early-study participants for whom identifying information was not collected. Percentages were calculated among participants with available data for each variable.

^d^ P<.05.

**Table 5. Sociodemographic and Clinical Associations With Perceived Benefits of Cardiac Rehabilitation (N=119)**

|  | | **All, n=119** | **Lower Chances of Another Cardiac Event (N=119)** | | | | | | | | **Prevent Condition from Worsening (N=119)** | | | | | | | **Improve Ability to Perform Daily Activities (N=119)** | | | | **Improve Sense of Wellbeing**  **(N=119)** | | | **Understand Condition**  **(N=119)** | | | | |
| --- | --- | --- | --- | --- | --- | --- | --- | --- | --- | --- | --- | --- | --- | --- | --- | --- | --- | --- | --- | --- | --- | --- | --- | --- | --- | --- | --- | --- | --- |
|  |  |  | **No**  **(n=11)** | **Yes**  **(n=108)** | | | | **P-value^a^** | | | **No**  **(n=8)** | | | **Yes**  **(n=111)** | | **P-value^a^** | | **No**  **(n=4)** | **Yes**  **(n=115)** | | **P-value^a^** | **No**  **(n=22)** | **Yes**  **(n=97)** | **P-value^a^** | **No**  **(n=7)** | | **Yes**  **(n=112)** | | **P-value^a^** |
| **Age, yr^b^** | | **64.2 (13.0)** | **64.2 (11.6)** | **108 (13.2)** | | | | **0.51** | | | **58.3 (13.5)** | | | **64.7 (13.0)** | | **0.18** | | **60 (5.7)** | **64.4 (13.2)** | | **0.51** | **65.2 (11.6)** | **64.0 (13.4)** | **0.71** | **62.4 (13.2)** | | **64.4 (13.1)** | | **0.71** |
| **Sex** | |  |  |  | | | |  | | |  | | |  | |  | |  |  | |  |  |  |  |  | |  | |  |
|  | Women | 36.1 (43) | 11.6 (5) | 88.4 (38) | | | | 0.50 | | | 7.0  (3) | | | 93.0 (40) | | 0.93 | | 2.3  (1) | 97.7 (42) | | 0.64 | 23.3 (10) | 76.7 (33) | 0.31 | 4.7  (2) | | 95.3 (41) | | 0.67 |
|  | Men | 63.9 (76) | 7.9  (6) | 92.1 (70) | | | |  |  |  | 6.6  (5) | | | 93.4 (71) | |  |  | 3.9  (3) | 96.1 (73) | |  | 15.8 (12) | 84.2 (64) |  | 6.6  (5) | | 93.4 (71) | |  |
| **Race** | |  |  |  | | | |  | | |  | | |  | |  | |  |  | |  |  |  |  |  | |  | |  |
|  | White | 52.1 (62) | 11.3 (7) | 88.7 (55) | | | | 0.42 | | | 8.1  (5) | | | 91.9 (57) | | 0.54 | | 3.2  (2) | 96.8 (60) | | 0.93 | 25.8 (16) | 74.2 (46) | **0.03^d^** | 6.5  (4) | | 93.5 (58) | | 0.78 |
|  | Non-White | 47.9 (57) | 7.0  (4) | 93.0 (53) | | | |  |  |  | 5.3  (3) | | | 94.7 (54) | |  |  | 3.5  (2) | 96.5 (55) | |  | 10.5  (6) | 89.5 (51) |  | 5.3  (3) | | 94.7 (54) | |  |
| **Education** | |  |  |  | | | |  | | |  | | |  | |  | |  |  | |  |  |  |  |  | |  | |  |
|  | High school or less | 26.1 (31) | 6.4  (2) | 93.6 (29) | | | 0.53 | | | | 0  (0) | | | 100 (31) | | | 0.08 | 3.2  (1) | 96.8 (30) | | 0.96 | 12.9  (4) | 87.1 (27) | 0.35 | 3.2  (1) | | 96.8 (30) | | 0.47 |
|  | Greater than high school | 74.0 (88) | 10.2 (9) | 89.8 (79) | | |  |  |  |  | 9.1  (8) | | | 90.9 (80) | | |  | 3.4  (3) | 96.6 (85) | |  | 20.5 (18) | 79.5 (70) |  | 6.8  (6) | | 93.2 (82) | |  |
| **Marital status^c^** | |  |  |  | | | | | | | | | | | | | | | | |  |  |  |  |  | |  | |  |
|  | Married | 52.0 (53) | 5.7  (3) | 94.3 (50) | | 0.62 | | | | 3.8 (2) | | | 96.2 (51) | | 0.20 | | | 1.9  (1) | 98.1 (52) | | 0.27 | 20.8 (11) | 79.2 (42) | 0.84 | 9.4  (5) | | 90.6 (48) | | 0.29 |
|  | Unmarried | 48.0 (49) | 8.2  (4) | 91.8 (45) | |  |  |  |  | 10.2 (5) | | | 89.8 (44) | |  |  |  | 6.1  (3) | 93.9 (46) | |  | 22.4 (11) | 77.6 (38) |  | 4.1  (2) | | 95.9 (47) | |  |
| **Employment status^c^** | |  |  |  | | | | | | | | | | | | | | | | |  |  |  |  |  | |  | |  |
|  | Full-time/ self-employed | 24.5 (25) | 12.0 (3) | 88.0 (22) | | 0.24 | | | | 16.0 (4) | | | 84.0 (21) | | **0.04^d^** | | | 4.0  (1) | 96.0 (24) | | 0.98 | 20.0  (5) | 80.0 (20) | 0.83 | 12.0 (3) | | 88.0 (22) | | 0.24 |
|  | Other | 75.5 (77) | 5.2  (4) | 94.8 (73) | |  |  |  |  | 3.9  (3) | | | 96.1 (74) | |  |  |  | 3.9  (3) | 96.1 (74) | |  | 22.1 (17) | 77.9 (60) |  | 5.2  (4) | | 94.8 (73) | |  |
| **Health insurance^c^** | |  |  |  | | | | | | | | | | | | | | | | |  |  |  |  |  | |  | |  |
|  | Private/ other | 61.9 (63) | 6.3  (4) | 93.7 (59) | | 0.79 | | | | 9.5  (6) | | | 90.5 (57) | | 0.18 | | | 4.8  (3) | 95.2 (60) | | 0.58 | 19.0 (12) | 81.0 (51) | 0.43 | 4.8  (3) | | 95.2 (60) | | 0.29 |
|  | Medicare/Medicaid | 38.2 (39) | 7.7  (3) | 92.3 (36) | |  |  |  |  | 2.6  (1) | | | 97.4 (38) | |  |  |  | 2.6  (1) | 97.4 (38) | |  | 25.6 (10) | 74.4 (29) |  | 10.3 (4) | | 89.7 (35) | |  |
| Number of prior hospitalizations^c^ | | 3 (2-5) | 3 (1-7) | 3 (2-5) | 0.80 | | | | 3 (2-5) | | | 3 (2-5) | | | 0.97 | | | 2 (1-7) | 3 (2-5) | 0.51 | | 3 (2-5) | 3 (2-5) | 0.74 | | 3 (1-4) | 3 (2-5) | 0.35 | |
| Sum of qualifying diagnoses^c^ | | 2 (1-2) | 2 (1-3) | 2 (1-2) | 0.56 | | | | 2 (1-2) | | | 2 (1-2) | | | 0.76 | | | 1 (1-3) | 2 (1-2) | 0.34 | | 2 (1-3) | 2 (1-2) | 0.27 | | 21 (1-2) | 2 (1-2) | 0.60 | |
| **Type of qualifying diagnosis^c^** | |  |  |  |  | | | |  | | |  | | |  | | |  |  |  | |  |  |  | |  |  |  | |
|  | Medical diagnosis | 33.3 (34) | 2.9  (1) | 97.1 (33) | 0.36 | | | | 2.9  (1) | | | 97.1 (33) | | | 0.06 | | | 8.8  (3) | 91.2 (31) | 0.19 | | 17.6  (6) | 82.4 (28) | 0.70 | | 2.9  (1) | 97.1 (33) | 0.36 | |
|  | Procedural diagnosis | 13.7 (14) | 14.3 (2) | 85.7 (12) |  |  |  |  | 21.4 (3) | | | 78.6 (11) | | |  |  |  | 0  (0) | 100 (14) |  |  | 28.6  (4) | 71.4 (10) |  |  | 14.3 (2) | 85.7 (12) |  |  |
|  | Both diagnoses | 52.9 (54) | 7.4  (4) | 92.6 (50) |  |  |  |  | 5.6  (3) | | | 94.4 (51) | | |  |  |  | 1.9  (1) | 98.1 (53) |  |  | 22.2 (12) | 77.8 (42) |  |  | 7.4  (4) | 92.6 (50) |  |  |
| **Prior cardiac rehabilitation^c^** | |  |  |  |  | | | |  | | |  | | |  | | |  |  |  | |  |  |  | |  |  |  | |
|  | No prior rehab | 69.6 (71) | 2.8  (2) | 97.2 (69) | **0.01^d^** | | | | 4.2  (3) | | | 95.8 (68) | | | 0.11 | | | 5.6  (4) | 94.4 (67) | 0.18 | | 18.3 (13) | 81.7 (58) | 0.23 | | 4.2  (3) | 95.8 (68) | 0.11 | |
|  | Prior cardiac rehab | 30.4 (31) | 16.1 (5) | 83.9 (26) |  |  |  |  | 12.9 (4) | | | 87.1 (27) | | |  |  |  | 0  (0) | 100 (31) |  |  | 29.0  (9) | 71.0 (22) |  |  | 12.9 (4) | 87.1 (27) |  |  |

^a^ P values were calculated using chi-square or Fisher exact tests for categorical variables, independent t tests for normally distributed continuous variables, and Mann-Whitney U tests for nonnormally distributed continuous variables, as appropriate.

^b^ Continuous variables are presented as mean (SD), and categorical variables are presented as % (n), unless otherwise indicated.

^c^ Denominators vary because of item-level survey nonresponse and missing electronic medical record-derived variables for early-study participants for whom identifying information was not collected. Percentages were calculated among participants with available data for each variable.

^d^ P<.05.

**Table 6. Sociodemographic and Clinical Associations With Barriers to Attending Cardiac Rehabilitation (N=134)**

|  | | **All, n=134** | **Cost**  **(N=134)** | | | **Travel**  **(N=134)** | | | **Absence from Work (N=134)** | | | **Paperwork**  **(N=134)** | | | **Time (tight schedule) (N=134)** | | | | | |
| --- | --- | --- | --- | --- | --- | --- | --- | --- | --- | --- | --- | --- | --- | --- | --- | --- | --- | --- | --- | --- |
|  |  |  | **No**  **(n=90)** | **Yes**  **(n=44)** | **P-value^a^** | **No**  **(n=61)** | **Yes**  **(n=73)** | **P-value^a^** | **No**  **(n=105)** | **Yes**  **(n=29)** | **P-value^a^** | **No**  **(n=121)** | **Yes**  **(n=13)** | **P-value^a^** | **No**  **(n=92)** | | | **Yes**  **(n=42)** | | **P-value^a^** |
| **Age, yr^b^** | | **64.2 (13.2)** | **65.0 (13.4)** | **62.3 (12.8)** | **0.36** | **63.5 (13.9)** | **64.8 (12.6)** | **0.55** | **66.2 (13.0)** | **57.2 (11.4)** | **0.001^d^** | **63.9 (13.5)** | **67.5 (8.7)** | **0.34** | **64.8 (12.9)** | | | **63.0 (13.8)** | | **0.45** |
| **Sex** | |  |  |  |  |  |  |  |  |  |  |  |  |  |  | | |  | |  |
|  | Women | 37.3 (50) | 74.0 (37) | 26.0 (13) | 0.19 | 36.0 (18) | 64.0 (32) | 0.09 | 88.0 (44) | 12.0 (6) | **0.04^d^** | 88.0 (44) | 12.0 (6) | 0.49 | 74.0 (37) | | | 26.0 (13) | | 0.30 |
|  | Men | 62.7 (84) | 63.1 (53) | 36.9 (31) |  | 51.2 (43) | 48.8 (41) |  | 72.6 (61) | 27.4 (23) |  | 91.7 (77) | 8.3 (7) |  | 65.5 (55) | | | 34.5 (29) | |  |
| **Race** | |  |  |  |  |  |  |  |  |  |  |  |  |  |  | | |  | |  |
|  | White | 56.7 (76) | 68.4 (52) | 31.6 (24) | 0.72 | 51.3 (39) | 48.7 (37) | 0.12 | 76.3 (58) | 23.7 (18) | 0.51 | 93.4 (71) | 6.6 (5) | 0.16 | 64.5 (49) | | | 35.5 (27) | | 0.23 |
|  | Non-White | 43.3 (58) | 65.5 (38) | 34.5 (20) |  | 37.9 (22) | 62.1 (36) |  | 81.0 (47) | 19.0 (11) |  | 86.2 (50) | 13.8 (8) |  | 74.1 (43) | | | 25.9 (15) | |  |
| **Education** | |  |  |  |  |  |  |  |  |  |  |  |  |  |  | | |  | |  |
|  | High school or less | 26.1 (35) | 57.1 (20) | 42.9 (15) | 0.14 | 48.6 (17) | 51.4 (18) | 0.67 | 80.0 (28) | 20.0 (7) | 0.78 | 91.4 (32) | 8.57 (3) | 0.79 | 68.6 (24) | | | 31.4 (11) | | 0.99 |
|  | Greater than high school | 73.9 (99) | 70.7 (70) | 29.3 (29) |  | 44.4 (44) | 55.6 (55) |  | 77.8 (77) | 22.2 (22) |  | 89.9 (89) | 10.1 (10) |  | 68.7 (68) | | | 31.3 (31) | |  |
| **Marital status^c^** | |  |  |  |  |  |  |  |  |  |  |  |  |  |  | | |  | |  |
|  | Married | 53.3 (64) | 75.0 (48) | 25.0 (16) | 0.06 | 39.1 (25) | 60.9 (39) | 0.16 | 81.3 (52) | 18.8 (12) | 0.29 | 90.6 (58) | 9.4 (6) | 0.58 | 67.2 (43) | | | 32.8 (21) | | 0.90 |
|  | Unmarried | 46.7 (56) | 58.9 (33) | 41.1 (23) |  | 51.8 (29) | 48.2 (27) |  | 73.2 (41) | 26.8 (15) |  | 87.5 (49) | 12.5 (7) |  | 66.1 (37) | | | 33.9 (19) | |  |
| **Employment status^c^** | |  |  |  |  |  |  |  |  |  |  |  |  |  |  | | |  | |  |
|  | Full-time/self-employed | 27.5 (33) | 72.7 (24) | 27.3  (9) | 0.25 | 63.6 (21) | 36.4 (12) | **0.01^d^** | 48.5 (16) | 51.5 (17) | **<0.001^d^** | 90.9 (30) | 9.1 (3) | **0.01^d^** | 51.5 (17) | | | 48.5 (16) | | **0.03^d^** |
|  | Other | 72.5 (87) | 65.5 (57) | 34.5 (30) |  | 37.9 (33) | 62.1 (54) |  | 88.5 (77) | 11.5 (10) |  | 88.5 (77) | 11.5 (10) |  | 72.4 (63) | | | 27.6 (24) | |  |
| **Health insurance^c^** | |  |  |  |  |  |  |  |  |  |  |  |  |  |  | | |  | |  |
|  | Private/other | 62.5 (75) | 62.7 (47) | 37.3 (28) | 0.14 | 53.3 (40) | 46.7 (35) | **0.02^d^** | 68.0 (51) | 32.0 (24) | **0.001^d^** | 94.7 (71) | 5.3 (4) | **0.01^d^** | 64.0 (48) | | | 36.0 (27) | | 0.42 |
|  | Medicare/ Medicaid | 37.5 (45) | 75.6 (34) | 24.1 (11) |  | 31.1 (14) | 68.9 (31) |  | 93.3 (42) | 6.67 (3) |  | 80.0 (36) | 20.0 (9) |  | 71.1 (32) | | | 28.9 (13) | |  |
| Number of Prior Hospitalizations^c^ | | 3 (2-5) | 3 (2-4) | 3 (2-6) | 0.36 | 3 (1-4) | 3 (2-6) | **0.02^d^** | 3 (2-5) | 3 (2-4) | 0.29 | 3 (2-5) | 3 (2-4) | 0.88 | 3 (2-5) | | | 3 (2-4) | | 0.49 |
| Sum of Qualifying Diagnoses^c^ | | 2 (1-2) | 2 (1-2) | 2 (1-2) | 0.63 | 2 (1-3) | 2 (1-2) | 0.69 | 2 (1-2) | 2 (1-2) | 0.84 | 2 (1-2) | 2 (1-2) | 0.82 | 2 (1-3) | | | 2 (1-2) | | 0.43 |
| **Type of Qualifying Diagnosis^c^** | |  |  |  |  |  |  |  |  |  |  |  |  |  |  | | |  | |  |
|  | Medical diagnosis | 33.3 (40) | 52.5 (21) | 47.5 (19) | **0.046^d^** | 37.5 (15) | 62.5 (25) | 0.47 | 75.0 (30) | 25.0 (10) | 0.73 | 87.5 (35) | 12.5 (5) | 0.91 | 57.5 (23) | | | 42.5 (17) | | 0.31 |
|  | Procedural diagnosis | 15.8 (19) | 73.7 (14) | 26.3  (5) |  | 52.6 (10) | 47.4 (9) |  | 84.2 (16) | 15.8 (3) |  | 89.5 (17) | 10.5 (2) |  | 68.4 (13) | | | 31.6 (6) | |  |
|  | Both diagnoses | 50.8 (61) | 75.4 (46) | 24.6 (15) |  | 47.5 (29) | 52.5 (32) |  | 77.0 (47) | 23.0 (14) |  | 90.2 (55) | 9.84 (6) |  | 72.1 (44) | | | 27.9 (17) | |  |
| **Prior Cardiac Rehabilitation^c^** | |  |  |  |  |  |  |  |  |  |  |  |  |  |  | | |  | |  |
|  | No prior rehab | 65.8 (79) | 67.1 (53) | 32.9 (26) | 0.89 | 49.4 (39) | 50.6 (40) | 0.18 | 74.7 (59) | 25.3 (20) | 0.31 | 89.9 (71) | 10.1 (8) | 0.73 | | 70.9 (56) | 29.1 (23) | | 0.17 | |
|  | Prior cardiac rehab | 34.2 (41) | 68.3 (28) | 31.7 (13) |  | 36.6 (15) | 63.4 (26) |  | 82.9 (34) | 17.1 (7) |  | 87.8 (36) | 12.2 (5) |  |  | 58.5 (24) | 41.5 (17) | |  |  |

^a^ P values were calculated using chi-square or Fisher exact tests for categorical variables, independent t tests for normally distributed continuous variables, and Mann-Whitney U tests for nonnormally distributed continuous variables, as appropriate.

^b^ Continuous variables are presented as mean (SD), and categorical variables are presented as % (n), unless otherwise indicated.

^c^ Denominators vary because of item-level survey nonresponse and missing electronic medical record-derived variables for early-study participants for whom identifying information was not collected. Percentages were calculated among participants with available data for each variable.

^d^ P<.05.
